# RNA-seq analysis of gene expression profiles in isolated stria vascularis from wild-type and Alport mice reveals key pathways underling Alport strial pathogenesis

**DOI:** 10.1371/journal.pone.0237907

**Published:** 2020-08-21

**Authors:** Brianna Dufek, Daniel T. Meehan, Duane Delimont, Kevin Wilhelm, Gina Samuelson, Ross Coenen, Jacob Madison, Edward Doyle, Brendan Smyth, Grady Phillips, Michael Anne Gratton, Dominic Cosgrove

**Affiliations:** 1 Boys Town National Research Hospital, Omaha, NE, United States of America; 2 Department of Otolaryngology, Wake Forest School of Medicine, Washington University, Saint Louis, MO, United States of America; University of California San Francisco, UNITED STATES

## Abstract

Previous work demonstrates that the hearing loss in Alport mice is caused by defects in the stria vascularis. As the animals age, progressive thickening of strial capillary basement membranes (SCBMs) occurs associated with elevated levels of extracellular matrix expression and hypoxia-related gene and protein expression. These conditions render the animals susceptible to noise-induced hearing loss. In an effort to develop a more comprehensive understanding of how the underlying mutation in the COL4A3 gene influences homeostasis in the stria vascularis, we performed vascular permeability studies combined with RNA-seq analysis using isolated stria vascularis from 7-week old wild-type and Alport mice on the 129 Sv background. Alport SCBMs were found to be less permeable than wild-type littermates. RNA-seq and bioinformatics analysis revealed 68 genes were induced and 61 genes suppressed in the stria from Alport mice relative to wild-type using a cut-off of 2-fold. These included pathways involving transcription factors associated with the regulation of pro-inflammatory responses as well as cytokines, chemokines, and chemokine receptors that are up- or down-regulated. Canonical pathways included modulation of genes associated with glucose and glucose-1-PO4 degradation, NAD biosynthesis, histidine degradation, calcium signaling, and glutamate receptor signaling (among others). In all, the data point to the Alport stria being in an inflammatory state with disruption in numerous metabolic pathways indicative of metabolic stress, a likely cause for the susceptibility of Alport mice to noise-induced hearing loss under conditions that do not cause permanent hearing loss in age/strain-matched wild-type mice. The work lays the foundation for studies aimed at understanding the nature of strial pathology in Alport mice. The modulation of these genes under conditions of therapeutic intervention may provide important pre-clinical data to justify trials in humans afflicted with the disease.

## Introduction

The stria vascularis is a highly specialized tissue lining the lateral wall comprised of marginal cells at the luminal surface that form basolateral infolds with intermediate cells and basal cells that are attached to the spiral ligament fibrocytes. Specialized pericytes and endothelial cells along with the strial capillary basement membranes maintain a tight fluid barrier of the strial capillaries [1]. The stria functions through various channels and transporters to maintain the endocochlear potential (EP) of the endolymph in the scala media of the cochlear duct. This EP is what drives the depolarization of cochlear hair cells when the mechanotransduction channels in the hair cell stereocilia open in response to movement of the basilar membrane. Thus, defects in strial function result in hearing loss. Alport mice are used as a model to study strial dysfunction.

Alport syndrome is characterized by delayed onset glomerular disease associated with progressive hearing loss [2]. The syndrome results from mutations in basement membrane type IV collagen genes COL4A3, COL4A4 (autosomal recessive Alport syndrome, [3, 4], and COL4A5 [X-linked Alport syndrome, [5]]. In the glomerulus of the kidney, the glomerular basement membranes, which are initially thinner than that of normal GBM owing to the lack of the type IV collagen α3/4/5 network, become progressively and irregularly thickened and thinned with multiple laminations of electron dense areas. This diagnostic phenotype, which is linked to the onset and progression of proteinuria, ultimately culminates in focal segmental glomerulosclerosis associated with renal failure.

In the inner ear we previously suggested basement membrane thickening occurs specifically in the strial capillary basement membranes (SCBMs) based on electron microscopy analysis of basement membrane thickening in 7–9 week-old autosomal Alport mice [6]. This was associated with the accumulation of basement membrane proteins, including laminins, entactin, and type IV collagen, and elevated expression of hypoxia-associated genes and matrix metalloproteinases [7, 8].

Alport mice showed permanent ABR threshold shifts after a noise exposure that was insufficient to cause permanent threshold shifts in age/strain matched wild-type mice [8]. These data strongly suggest that the stria vascularis, which shows a loss of metabolic homeostasis, is the source of pathologic hearing loss in the Alport mouse model.

In order to further our understanding of the nature of the changes that occur in the Alport stria vascularis compared to healthy wild-type stria, we verified that the thickening of the SCBM negatively influenced vascular permeability, which may account for the metabolic stress. We then performed RNA-seq analysis of strial RNA from wild-type and Alport mice at an age when SCBM thickening is apparent. The data confirm that the stria vascularis in the 7-week-old 129 Sv Alport mouse model is injured and dysfunctional. Moreover, the data provide new information informing whether current or planned pre-clinical trials of novel therapeutics can restore normal strial homeostasis.

## Methods

### Mice

129 Sv autosomal Alport mice were developed in the Cosgrove lab [9]. All mice were on a pure 129 Sv genetic background and maintained in house. Lab diet was Teklad Envigo diet # 7912.15. All procedures involving animals were conducted in accordance of an approved IACUC protocols at both sites (Boys Town National Research Hospital and Saint Louis University) and consistent with the NIH guide for the care and use of laboratory animals. Every effort was made to minimize usage as well as minimize any pain or distress. Both males and females were utilized. Animals were housed in groups with in rooms with a 14/10 hour light/dark cycle.

### Strial microdissection

A detailed procedure for strial microdissection was described previously [7]. The temporal bones were harvested from non-transcardially perfused mice following cervical dislocation (within two minutes) and transferred to ice-cold HBSS buffer in a specialized petri dish. Both stria were microdissected within 10 minutes and transferred to TriZol for later RNA isolation. The stria from eight wild-type and eight Alport mice were combined for a single analysis and the experiment was performed two independent times.

### RNA-seq analysis

Microdissected striae were lysed in Trizol® (Ambion®, Carlsbad, CA) and RNA isolated from the aqueous phase using PureLink® RNA Mini Kit (Ambion®). An RNA Quality Number (RQN) was determined for each sample using a Fragment Analyzer™ Automated CE System (Advanced Analytical Technologies, Inc. Ames, IA). Samples with RQN’s of ≥ 8 were used in SMART-Seq®v4 Ultra® (Takara Bio USA, Inc.). cDNA synthesis and libraries generated utilizing Nextera™ XT DNA Library Preparation Kit (Illumina® San Diego, CA). RNA seq analysis was performed using the Illumina® NextSeq™ 500 system (San Diego, CA). The data was analyzed using Ingenuity Pathway Analysis software (QIAGEN Bioinformatics) by the University of Nebraska Medical Center bioinformatics core facility. This is a classification of the data into categories and then sub-categories. For example, major categories include canonical pathways, transcriptional regulators, disease functions, and toxicity functions. These are further broken down to sub-categories based on disease functions, these include immunological disease, metabolic disease endocrine disorders and organismal injury. For each sub-category the molecules that are significantly modulated and the degree of that modulation are listed by the software based on a two-fold cut-off for up- or down-regulation. Because this categorization has considerable redundancy, given that the program was written as for broad-spectrum analysis, we present general categories that the literature suggests reflect the underlying causes of strial pathology, which is not a category probed by the software because it is too specific [1, 10, 11]. This is the data presented in Tables 1–4. The unbiased nature of this approach is reflected in the fact that >80% of these genes have never been ascribed as functionally important in the stria vascularis. Standard deviations are provided for two independent RNA-seq experiments. The complete set of raw data can be accessed at https://www.ncbi.nlm.nih.gov/sra/?term=PRJNA602720.

**Table 1:** Dysregulation of genes involved in cell morphology.

Gene Name	Protein	Function	Fold Change Alp/WT	
			Average	Std Dev
Card11	Caspase recruitment domain-containing protein 11	NF-kappa-B activation	20.00	11.31
Gata1	Erythroid transcription factor	transcription factor	12.00	8.49
Il17f	Interleukin-17F	pro-inflammatory cytokine	9.50	5.66
Klf1	Krueppel-like factor 1	transcription factor	9.50	1.41
Prss16	Thymus-specific serine protease	protease	8.83	1.18
Lta	Lymphotoxin-alpha	TNF-B in cochlea	7.00	2.36
Dcdc2a	Doublecortin domain-containing protein 2	tubulin polymerization	6.75	3.18
***Tnf***	***Tumor necrosis factor***	***NF-kappa-B activation*, *cytokine***	***6*.*60***	***3*.*11***
Hamp2	Hepcidin-2	iron storage/SMAD signaling	6.43	1.82
Ntrk1	High affinity nerve growth factor receptor	MAPK activation	5.00	1.41
Dlx2	Homeobox protein DLX-2	transcriptional activator	4.00	0.00
Bdkrb2	B2 bradykinin receptor	ERK 1/2 activation	3.83	0.24
Efna3	Ephrin-A3	tyrosine kinase	3.59	1.22
***Cldn24***	***Claudin 24***	***cell adhesion***	***3*.*57***	***1*.*5***
Pvalb	Parvalbumin	calcium signaling	3.55	2.70
Ly9	T-lymphocyte surface antigen Ly-9	Regulates IL-17 production	3.50	2.50
Nefh	Neurofilament heavy polypeptide	Neurofilament	3.47	2.91
Slc8a1	Sodium/calcium exchanger 1	calcium signaling	3.33	1.41
Tulp1	Tubby-related protein 1	Cell survival	3.27	1.03
Prkg2	cGMP-dependent protein kinase 2	Serine/threonine kinase	3.00	2.36
Pou2af1	POU domain class 2-associating factor 1	transcription factor	3.00	0.61
***Spink5***	***Serine protease inhibitor Kazal-type 5***	***Serine protease inhibitor***	***2*.*94***	***0*.*11***
Ccl7	C-C motif chemokine 7	chemokine	2.79	0.34
Cd79b	B-cell antigen receptor complex-associated protein beta chain	B lymphocyte antigen receptor	2.72	1.02
Ccr5	C-C chemokine receptor type 5	chemokine	2.71	2.14
Fgf2	Fibroblast growth factor 2	development	2.69	0.11
Col10a1	Collagen alpha-1(X) chain	Cell matrix	2.67	0.94
Gpr3	G-protein coupled receptor 3	G-protein coupled receptor activity.	2.63	0.53
Nefm	Neurofilament medium polypeptide	Neurofilament	2.62	0.87
Bco1	Beta, beta-carotene 15,15'-dioxygenase	metabolism	2.45	1.16
Prkcg	Protein kinase C gamma type	Serine/threonine kinase	2.42	0.24
Cd247	T-cell surface glycoprotein CD3 zeta chain	signal transduction	2.41	1.58
Trpm2	Transient receptor potential cation channel subfamily M member 2	oxidative stress, activated calcium influx	2.41	0.04
Slc26a4	Pendrin	Sodium-independent transporter	2.25	0.06
Grid2	Glutamate receptor ionotropic, delta-2	neurotransmitter	2.19	0.39
Fgf21	Fibroblast growth factor 21	development	2.18	0.00
Iqgap3	IQ motif-containing GTPase-activating protein 3	actin cytoskeleton/adhesion	2.13	0.46
Bach2	Transcription regulator protein BACH2	transcription factor, activates NFkB	2.04	0.27
Fgf22	Fibroblast growth factor 22	Development	2.00	0.42
***Icam1***	***Icam1***	***Cell adhesion***	***1*.*74***	***0*.*31***
Jph1	Junctophilin-1	cell surface ion channels	-1.30	0.51
Clip3	CAP-Gly domain-containing linker protein 3	cytoplasmic linker protein	-2.08	0.48
Vcan	Versican core protein	large ECM chondroitin sulfate proteoglycan	-2.27	2.43
Elf4	ETS-related transcription factor Elf-4	transcription factor	-2.27	0.93
Igf2	Insulin-like growth factor II	WNT signaling	-2.27	1.50
Ncam2	Neural cell adhesion molecule 2	cell adhesion	-2.50	1.44
Grik2	Glutamate receptor ionotropic, kainate 2	neurotransmitter	-2.56	0.72
Slc4a4	Electrogenic sodium bicarbonate cotransporter 1	anion exchange	-2.56	1.31
Jph4	Junctophilin-4	cell surface ion channels	-2.63	2.42
Kcna5	Potassium voltage-gated channel subfamily A member 5	mediates potassium transport	-2.86	3.02
Slc17a8	Vesicular glutamate transporter 3	vesicular glutamate transporter	-3.03	2.20
Astn1	Astrotactin-1	Cell migration	-3.03	0.00
Pla2g10	Group 10 secretory phospholipase A2	Phospholipase	-3.23	1.56
Sez6	Seizure protein 6	neuronal membrane signaling	-3.33	1.89
Islr2	Immunoglobulin superfamily containing leucine-rich repeat protein 2		-3.45	0.29
***Spock1***	***Testican-1***	***Protease inhibitor***	***-3*.*52***	***0*.*34***
Fshb	Follitropin subunit beta	hormone	-3.57	0.38
Pou3f2	POU domain, class 3, transcription factor 2	transcription factor	-3.70	5.35
Sost	Sclerostin	WNT signaling	-3.70	5.35
Cntf	Ciliary neurotrophic factor	Cell survival	-5.00	7.25
Tnfrsf4	Tumor necrosis factor receptor superfamily member 4	NF-kappa-B activation	-5.00	1.75
Jph3	Junctophilin-3	cell surface ion channels	-5.26	7.48
Myoz1	Myozenin-1	actin cytoskeleton	-5.26	2.49
Muc2	Mucin-2	Secreted barrier glycoprotein	-6.67	1.78
Lag3	Lymphocyte activation gene 3 protein	immune function	-8.33	11.81
Gfi1	Zinc Finger Protein Gfi-1	transcription repressor	-8.33	0.00
Pspn	Persephin	GDNF family, protective	-10.00	15.00
**Nptx1**	**Neuronal pentraxin-1**	**mediates hypoxic-ischemic injury**	**-11.11**	**3.70**
Gjc2	Gap junction gamma-2 protein	gap junction	-11.11	14.81
Pou4f1	POU domain, class 4, transcription factor 1	transcription factor	-12.50	18.75
**Gfap**	**Glial fibrillary acidic protein**	**cytoskeleton**	**-16.67**	**19.44**
Mlc1	Membrane protein MLC1	homolog to KCNA1 (KIV1.1)	-25.00	12.50
**Reln**	**Reelin**	**Extracellular matrix serine protease-protective activities**	**-50.00**	**75.00**

**Table 2:** Dysregulation of genes associated with tissue injury and inflammation.

Gene Name	Protein	Function	Fold Change Alp/WT	
			Average	Std Dev
***Kcnip1***	***Kv channel-interacting protein 1***	***potassium transport***	***23*.*00***	***15*.*56***
Ccl9	C-C motif chemokine 9	chemokine	10.50	4.60
Il17f	Interleukin-17F	pro-inflammatory cytokine	9.50	5.66
Ros1	Proto-oncogene tyrosine-protein kinase ROS	tyrosine kinase activity	7.50	3.54
Sit1	Signaling threshold-regulating transmembrane adapter 1	suppressor	7.00	3.30
Tnf	Tumor necrosis factor	NF-kappa-B activation, cytokine	6.60	3.11
Mmp12	Macrophage metalloelastase	ECM protease	6.00	4.53
Il12b	Interleukin-12 subunit beta	cytokine	5.50	4.95
***Grem1***	***Gremlin-1***	***cytokine*, *BMP antagonist***	***4*.*50***	***2*.*12***
Kcnc2	Potassium voltage-gated channel subfamily C member 2	potassium channel	4.00	2.83
Has3	Hyaluronan synthase 3	ECM hyaluronan	3.67	1.41
Ipcef1	Interactor protein for cytohesin exchange factors 1	cell migration	3.57	1.01
Pvalb	Parvalbumin	calcium signaling	3.55	2.70
H2-DMb2	HLA class II histocompatibility antigen, DM beta chain	endosomal membrane	3.52	0.10
Slc8a1	Sodium/calcium exchanger 1	calcium signaling	3.33	1.41
Tulp1	Tubby-related protein 1	Cell survival	3.27	1.03
Ms4a1	B-lymphocyte antigen CD20	B cell activation	3.00	0.47
Prkg2	cGMP-dependent protein kinase 2	Serine/threonine kinase	3.00	2.36
Spink5	Serine protease inhibitor Kazal-type 5	Serine protease inhibitor	2.94	0.11
Ccl7	C-C motif chemokine 7	chemokine	2.79	0.34
Slc6a19	Sodium-dependent neutral amino acid transporter B(0)AT1	transporter	2.73	1.29
Cd79b	B-cell antigen receptor complex-associated protein beta chain	B lymphocyte antigen receptor	2.72	1.02
Ccr5	C-C chemokine receptor type 5	chemokine	2.71	2.14
Gpr3	G-protein coupled receptor 3	G-protein coupled receptor activity.	2.63	0.53
Nefm	Neurofilament medium polypeptide	Neurofilament	2.62	0.87
Satb2	DNA-binding protein SATB2	transcription factor	2.47	0.47
Prkcg	Protein kinase C gamma type	Serine/threonine kinase	2.42	0.24
Cd247	T-cell surface glycoprotein CD3 zeta chain	cell signaling	2.41	1.58
Mrvi1	Protein MRVI1	Protein complex formation	2.31	1.31
Grid2	Glutamate receptor ionotropic, delta-2	neurotransmitter	2.19	0.39
Otoa	Otoancorin	tectorial adhesion	2.17	0.33
Bach2	Transcription regulator protein BACH2	transcription factor, activates NFkB	2.04	0.27
Il27ra	Interleukin-27 receptor subunit alpha	immune response	1.98	0.03
Il2ra	Interleukin-2 receptor subunit alpha	immune response	1.24	0.02
Igf2	Insulin-like growth factor II	WNT signaling	-2.27	1.50
Grik2	Glutamate receptor ionotropic, kainate 2	neurotransmitter	-2.56	0.72
Slc4a4	Electrogenic sodium bicarbonate cotransporter 1	anion exchange	-2.56	1.31
Zap70	Tyrosine-protein kinase ZAP-70	immune response	-2.70	1.02
Kcna1	Potassium voltage-gated channel subfamily A member 1	potassium channel	-2.94	1.64
Chil3	Chitinase-like protein 3	inflammation	-2.94	2.42
Il12a	Interleukin-12 subunit alpha	cytokine	-3.13	2.93
Pla2g10	Group 10 secretory phospholipase A2	Phospholipase	-3.23	1.56
Ptch2	Protein patched homolog 2	hedgehog	-3.23	1.14
Sez6	Seizure protein 6	neuronal membrane signaling	-3.33	1.89
Stap1	Signal-transducing adaptor protein 1	Cell signaling	-3.33	1.33
Sost	Sclerostin	WNT signaling	-3.70	5.35
Clec9a	C-type lectin domain family 9 member A	activation receptor	-3.70	5.21
Ngp	Neutrophilic granule protein	inhibitor of cathepsin B (CTSB) activity	-3.70	1.78
Kcnip2	Kv channel-interacting protein 2	potassium transport	-4.00	5.60
Smtnl1	Smoothelin-like protein 1	Calmodulin binding protein	-4.00	1.92
Slc2a5	Solute carrier family 2, facilitated glucose transporter member 5	fructose transporter	-4.55	6.40
Cntf	Ciliary neurotrophic factor	Growth factor	-5.00	7.25
Tnfrsf4	Tumor necrosis factor receptor superfamily member 4	NF-kappa-B activation	-5.00	1.75
Slitrk3	SLIT and NTRK-like protein 3	Suppresses neurite outgrowth	-5.00	3.50
Myoz1	Myozenin-1	actin cytoskeleton	-5.26	2.49
Kcnc1	Potassium voltage-gated channel subfamily C member 1	potassium channel	-5.56	2.47
Muc2	Mucin-2	Secreted barrier glycoprotein	-6.67	1.78
Ccl3	C-C motif chemokine 3	chemokine	-8.33	4.17
Cdhr1	Cadherin-related family member 1	cell adhesion	-9.09	13.22
Nptx1	Neuronal pentraxin-1	mediates hypoxic-ischemic injury	-11.11	3.70
Tmem178	Transmembrane protein 178A	NF-kappa-B activation	-20.00	28.00
Mlc1	Membrane protein MLC1	homolog to KCNA1 (KIV1.1)	-25.00	12.50

**Table 3:** Hearing loss.

Gene Name	Protein	Function	Fold Change Alp/WT	
			Average	Std Dev
Fgf10	Fibroblast growth factor 10	growth factor activity	6.00	5.66
Slc25a21	Mitochondrial 2-oxodicarboxylate carrier	transporter	5.91	1.67
Col1a1	Collagen alpha-1(I) chain	Extracellular matrix	3.50	0.86
Slc26a4	Pendrin	Sodium-independent transporter	2.25	0.06
Tmc1	Transmembrane channel-like protein 1	probable ion channel	-2.33	0.27
Tomt	Transmembrane O-methyltransferase homolog	mechanotransduction	-2.86	1.31
Ocm	Oncomodulin	calcium ion-binding	-2.94	1.12
Slc17a8	Vesicular glutamate transporter 3	vesicular glutamate transporter	-3.03	2.20
Bdnf	Brain-derived neurotrophic factor	Growth factor	-4.00	0.00
Gfi1	Zinc Finger Protein Gfi-1	transcription repressor	-8.33	0.00
**Hearing**				
Otoa	Otoancorin	Adhesion molecule	2.17	0.33
Lhfpl5	LHFPL tetraspan subfamily member 5 protein	mechanotransduction	-3.13	0.98
Asic2	Acid-sensing ion channel 2	cation transport	-11.11	16.05
Grxcr2	Glutaredoxin domain-containing cysteine-rich protein 2	metabolism	-14.29	20.41

**Table 4:** Upstream regulators.

	Gene Name	Protein	Function	Fold Change Alp/WT	
				Average	Std Dev
**GATA2**					
	Gata1	Erythroid transcription factor	transcription factor	12.00	8.49
	Klf1	Krueppel-like factor 1	transcription factor	9.50	1.41
	Mcemp1	Mast cell-expressed membrane protein 1	Cell differentiation	4.60	2.55
	Cd177	Cd177 antigen	activates TNF-primed cells	4.00	3.64
	Clec4e	C-type lectin domain family 4 member E	cell signaling	4.00	1.41
	Slc35d3	Solute carrier family 35, D3	transmembrane transport	3.50	2.12
	Retnlg	Resistin-like gamma	Extracellular/receptor binding	2.71	0.44
	Spns3	Protein spinster homolog 3	transporter	2.69	0.65
	Ngp	Neutrophilic granule protein	inhibitor of cathepsin B (CTSB) activity	-3.70	1.78
	Hemgn	Hemogen	Cell proliferation	-5.00	7.00
	Ear2	V-ErbA-Related Protein 2	transcription factor	-5.00	7.00
	Gfi1	Zinc Finger Protein Gfi-1	transcription repressor	-8.33	0.00
	Mmrn1	Multimerin-1	Carrier protein	-16.67	22.22
**STAT1**					
	Tnf	Tumor necrosis factor	NF-kappa-B activation, cytokine	6.60	3.11
	Apol6	Apolipoprotein L6	lipid binding	5.00	1.41
	Slc8a1	Sodium/calcium exchanger 1	calcium signaling	3.33	1.41
	Chil3	Chitinase-like protein 3	Injury response	-2.94	2.42
**NFKBIA**					
	Tnf	Tumor necrosis factor	NF-kappa-B activation, cytokine	6.60	3.11
	Ccl7	C-C motif chemokine 7	chemokine	2.79	0.34
	Ccl3	C-C motif chemokine 3	chemokine	-8.33	4.17
**FOXA2**					
	Ccr5	C-C chemokine receptor type 5	chemokine	2.71	2.14
	Aldob	Fructose-bisphosphate aldolase B	gluconeogenesis	2.07	1.31
	Tnni1	Troponin I, slow skeletal muscle	Muscle contraction	-2.33	0.54
	Astn1	Astrotactin-1	Adhesion/cell migration	-3.03	0.00
	Saa3	Serum amyloid A-3 protein	inflammation	-5.00	3.00
	Muc2	Mucin-2	Secreted barrier glycoprotein	-6.67	1.78
**JUNB**					
	Tnf	Tumor necrosis factor	NF-kappa-B activation, cytokine	6.60	3.11
	Il12b	Interleukin-12 subunit beta	cytokine	5.50	4.95
	Ccl3	C-C motif chemokine 3	chemokine	-8.33	4.17
					

### Vascular permeability assay

**Fig 1:**
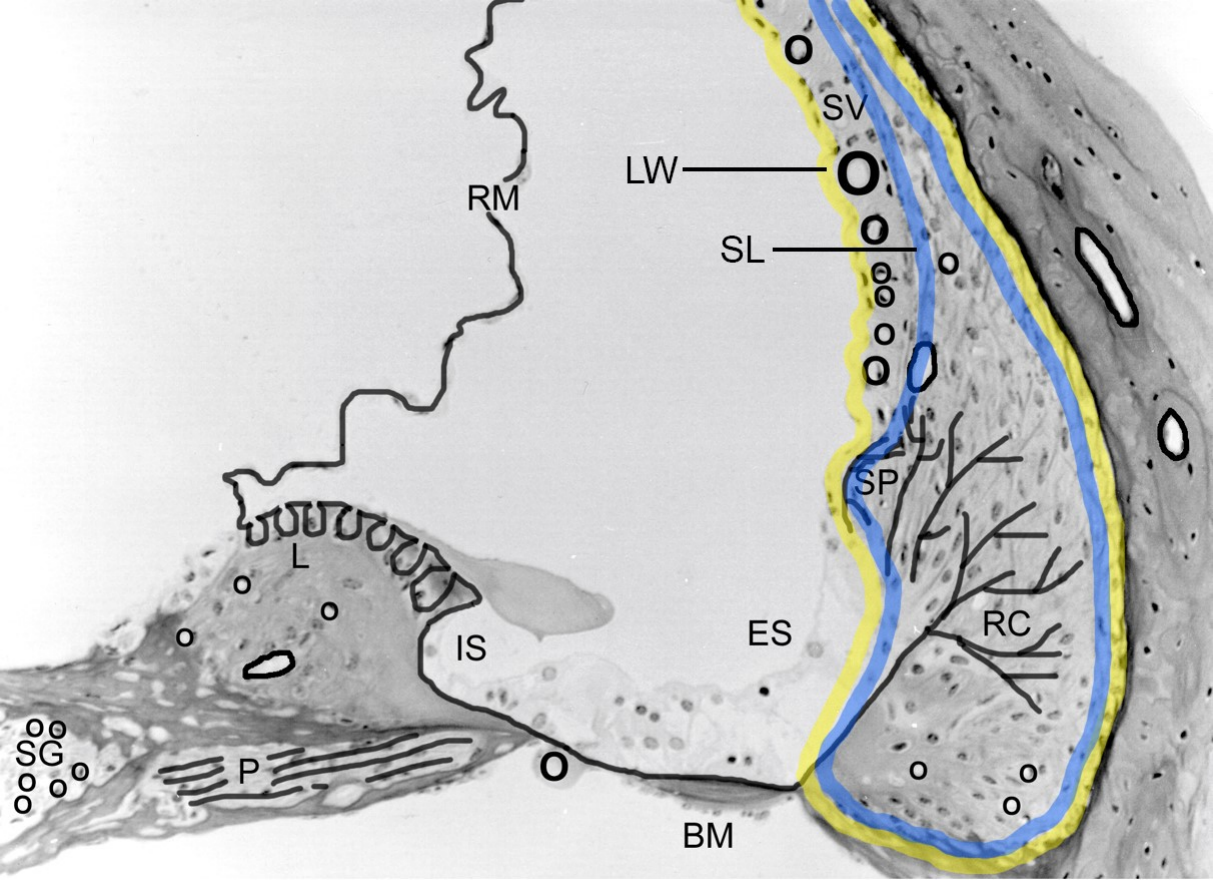
Mid-modiolar cross section of a mouse cochlea denoting basement membranes found in the membranous tissues. SG = spiral ganglion; P = perineurium; L = limbus; IS = inner sulcus; BM = basilar membrane; ES = external sulcus; RC = root cells; SP = spiral prominence; SV = stria vascularis; RM = Reissner’s membrane; SL = spiral ligament; LW = lateral wall. Note as per reference for Fig 2, the lateral wall is outlined in yellow and the spiral ligament is outlined in blue.

8.5 wk old Alport and WT mice (n = 16/genotype) were anesthetized (Avertin (0.4 mg/g BW, IP)). Each mouse received an intra-cardiac injection (200 μl) of unconjugated Rhodamine fluorescent dye (0.04% in sterile normal saline (NS)) followed by 3 minutes incubation. At 30 and 60 sec post-injection, the lips, nose and front paws were examined under UV light. If no fluorescence was noted, the experiment was aborted. If at 3 min post-injection, the lips, nose and front paws glowed, the mouse was transcardially perfused with 5ml 0.1M cacodylate to wash out the Rhodamine dye from the vasculature. The cochleae were isolated, perilymphatically perfused with, and then immersed in cold (4°C) 4% paraformaldehyde in 0.1M cacodylate buffer. Within 20 minutes, the lateral wall (LW, Fig 1) of both cochleae representing the stria vascularis and spiral ligament was microdissected, transferred into a 0.5 ml tube and frozen in liquid nitrogen. Since the stria vascularis tended to fragment if not attached to the lateral wall, In a subset of dissections, only the spiral ligament (SL, Fig 1) was collected to determine its contribution to lateral wall diffusion. The vascular permeability of the stria vascularis was the difference between that of the whole LW and the SL as shown in Fig 1. A negative control group (n = 7) was created using LW from mice that underwent intra-cardiac injection with 0.9% sterile NS in place of Rhodamine dye followed by isolation of the LW.

Each sample of two LWs or two SLs was iced and micro-homogenized (100 strokes) in 30 μl of sterile phosphate buffered saline (PBS). Fluorescence (550/580) was quantified in black 384 well low-volume plates. A standard curve was constructed for the unconjugated Rhodamine fluorescent dye (y = random fluorescence units (RFU) vs. x = concentration (%w/v)). Since at low levels of fluorescence the standard curve was nonlinear, the fluorescent dye concentration in each sample was determined by solving for the quadratic equation for its positive root (x1 or x2), expressed as %w/v. The permeability of the StV was determined by subtracting the mean fluorescence of the SL samples from the of the LW samples for each genotype.

### Immunofluorescence analysis

Mice were deeply anesthetized with ketamine and xylazine (300 mg/kg and 30 mg/kg, respectively, IP). The appropriate level of anesthesia was evaluated by loss of the hindquarter reflex after a pinch with thumb and index finger. If the indicated dose does not inhibit the reflex, an additional 20% of the anesthetic will be administered. Animals are euthanized by transcardial perfusion with PBS while under anesthesia. Cochleae were perfused with 4% paraformaldehyde and then decalcified overnight in 150mM EDTA on a rotator at 4°C. The samples were transferred to 15% sucrose for 1 hour and then into 30% sucrose for 2 hours. Samples were mounted in OCT and frozen at -80°C. Cochleae were sectioned at 8-μm and dipped in cold acetone. After air drying 2 hours, slides were rehydrated with 1X PBS. Cochleae sections used for collagen IV immunostaining were first denatured with acid urea (6M urea, 0.1M glycine, pH 3.5) for 1 hour at 4°C and then rinsed with 1X PBS. Slides were stained with one of the following antibodies: GFAP (Protein Tech, cat # 16825-1-AP) 1:200 in 1%BSA in 0.3% PBST (Triton X-100); NPTX1 (Protein Tech, cat# 20656-1-AP) 1:100 in 1%BSA in 0.3% PBST (Triton X-100); Reelin (ThermoFisher PA%-78413) 1:50 in 1%BSA in PBS; ICAM1 (R&D Systems Cat # AF796) 5 ug/ml in 7%non-fat dry milk in 0.3% PBST (Triton X-100) Kir4.1 1:200 in 1% BSA in 0.3% PBST (Triton X-100). For macrophage analysis in the stria, dual immunofluorescence analysis was performed using anti-desmin (pericyte marker, Abcam ab8592) and anti F4/80 (macrophage marker, GeneTex GTX26640) at 1:100 and 1:250 dilution, respectively. The experiment was performed using 4 independent animals for WT stria and 7 independent animals for Alport stria. Macrophages were counted blinded in three sections per cochlea separated by 40 μM.

### Confocal microscopy

Confocal images captured using a Leica TCS SP8 MP confocal imaging system, using a 63x NA: 1.4 oil or 10x NA: 0.3 objective. Final figures were assembled using Adobe Photoshop and Illustrator software (Adobe Systems, CA).

### Real-time RT-PCR analysis

cDNA was generated from the previously isolated Strial RNA (the same samples used for RNA-seq analysis), using Invitrogen SuperScript® VILO™ Master Mix (Thermo Fisher). Applied Biosystems™ TaqMan® Assays (Thermo Fisher) for GREM1 Mm00488615_s1, KCNIP1 Mm01189526_m1, CLDN 24 Mm01206808_s1, ICAM 1 Mm00516023_m1, SPINK 5 Mm00511522_m1, SPOCK 1 Mm00486393_m1 and TNF Mm0443258_m1 were run on a StepOnePlus Real-Time PCR System (Thermo Fisher). The data represent 7 independent wild-type samples and 9 independent Alport samples.

### Statistical analysis

For vascular permeability studies, a Kruskal-Wallis H-test followed by Dunn’s Multiple Comparison test was used to determine whether any tissue autofluorescence contributed to the measured rhodamine fluorescence. The presence of significant differences between the rhodamine fluorescence in the wild-type and Alport spiral ligament and lateral wall samples was determined using a 2-way ANOVA with factors of genotype and tissue followed by a Holm Sidak all pairwise post hoc analysis. The negative control group was removed from the ANOVA analysis because it contained only lateral wall tissue samples and thus violated a 2-way ANOVA assumption. Finally, a t-test was conducted to determine is the WT StV fluorescence differed significantly from that of the KO StV fluorescence. Significance was set at p<0.05. The statistics and resultant data graphs were conducted using Sigma Plot 13 (SYSTAT Software, San Jose, CA). Strial macrophages were counted and the data analyzed using a two-tailed students t-test. Real time RT-PCR data was analyzed using a two-tailed students t-test.

## Results

**Fig 2:**
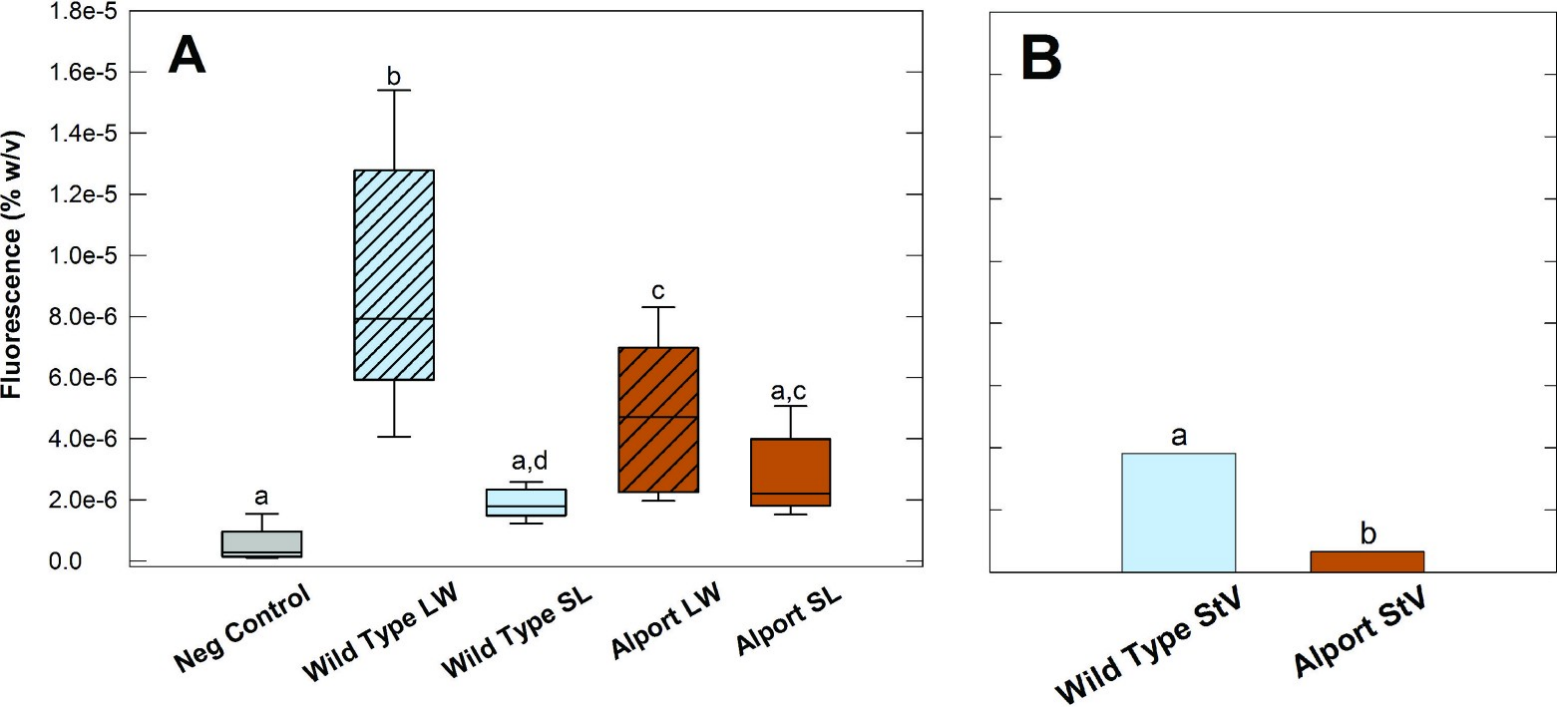
**(A)**. lower level of fluorescence in the Alport lateral wall tissue demonstrates a lower capillary permeability (ANOVA, F_(1,28)_ = 8.977, p = 0.006) in 8.5 week old Alport mice compared to wild-type littermates. The dark horizontal line within each box represents the median value while the top and bottom of the box denotes the 90^th^ and 10^th^ percentile of the data respectively. The line denotes the standard error. The lack of a significant permeability difference between the negative control lateral wall, wild-type and Alport spiral ligament tissues (Kruskal-Wallis, H = 14, p = 0.007, df = 4) suggests that the difference seen in the permeability of the cochlear lateral wall is attributable to reduced permeability of the strial vasculature and its thickened capillary basement membranes. (B). The height of the vertical bars is the difference between the median values of LW and SL fluorescence (black horizontal lines, Fig 2A). It represents the value of the StV fluorescence. The vascular permeability in the Alport StV is significantly lower than that in the wild-type StV. LW = lateral wall, SL = spiral ligament, Same letter denotes no significant difference.

The distribution of basement membranes (black solid lines) in a mid-modiolar cross-section of the mouse cochlea are shown in Fig 1. As a reference for Fig 2, we outlined the lateral wall in yellow and the spiral ligament in blue. Previous work showed significant thickening confined to the SCBMs in Alport mice at 7 weeks of age [6]. This same study showed that basement membranes in all other cochlear regions (Fig 1) did not vary significantly in the Alport mice compared to age-matched wild-type mice. Morphometric measures of SCBMs from 9 different wild-type mice and 9 different Alport mice between 8 and 9 weeks of age showed that the SCBMs were significantly thickened (59.7 +/- 19.1 nm for wild-type versus 98.7+/-38 nm for Alport SCBMs, [8]. We surmised that the thickening of the SCBMs might compromise the permeability of the strial capillaries. To test this, Rhodamine dye was injected intracardially into 8.5 week wild-type and Alport mice. The concentration of Rhodamine dye in cochlear lateral wall tissue after a transcardial flushing of the vasculature was quantified via fluorimetry against a standard curve. The results in Fig 2A show that the SCBMs in the stria of Alport mice are indeed less permeable than those in the Alport mice, presumably owing to the thickened SCBMs. Results of the Kruskal-Wallis (H = 14, p = 0.007, df = 4) and multiple comparison test (Dunn’s, p<0.05) showed that the level of fluorescence of the negative control lateral wall (LW; Fig 1) as well as that of the spiral ligament (SL; Fig 1) tissue from both wild-type and Alport cochlea were significantly lower than that present in the lateral wall tissue of both the wild-type and Alport mice. Further analysis revealed that a significant interaction existed between the genotype and tissue (ANOVA, F_(1,28)_ = 8.977, p = 0.006). Post hoc analysis revealed that the significance (Holm-Sidak, p<0.05) is due to the lower permeability of Rhodamine from capillaries to the surrounding lateral wall tissue in Alport versus wild-type mice. No difference (Holm-Sidak, p>0.05) was noted in the fluorescence of spiral ligament tissue in the two types of mice. Together, the lateral wall and spiral ligament data indicate that the lower vascular permeability in the Alport lateral wall is due to the accumulation of basement membrane proteins resulting in thickened strial capillaries with dysfunctional endothelial and pericyte cells. To test this conclusion, StV permeability was determined from the difference in the median level of fluorescence between LW and SL tissues for each genotype. Fig 2B shows that the permeability of the StV in the Alport mouse is significantly less (t-test, p<0.001) than that of the wild-type mice.

**Fig 3:**
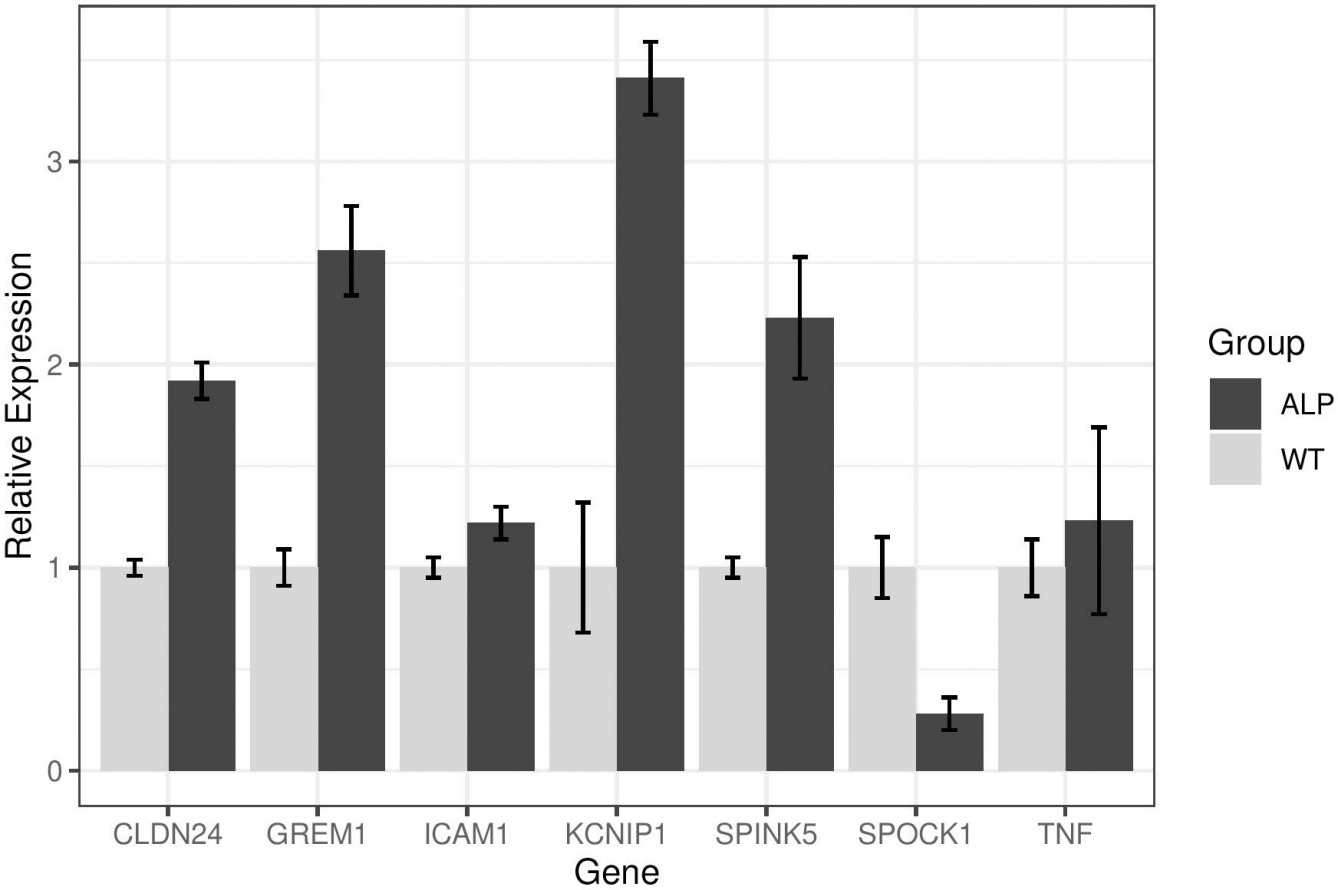
Confirmation of RNA-seq findings using real time RT-PCR. Strial RNA from seven wild-type mice and nine Alport mice were analyzed independently for the seven indicated transcripts by real time RT-PCR. All confirmed RNA-seq results except for Tnf-α. This may be due to taq-man probes only spanning a single exon, possibly missing alternatively spliced isoforms. Data is presented as the average with standard deviations. Data was analyzed using two-tailed students t-test. *p<0.05.

We presumed that reduced permeability might compromise strial function. To derive a clearer understanding of the health of the Alport stria vascularis we performed RNA-seq analysis using microdissected stria from 7-week-old wild-type and Alport mice. The stria vascularis was microdissected from (eight each) 7-week-old wild-type and Alport mice. The strial cDNA was sequenced and the data analyzed using Ingenuity Pathway Analysis software (QIAGEN Bioinformatics). The experiment was performed twice. Only genes that were consistently up- or down-regulated in both independent experiments by at least two-fold are presented. We broke our analysis into four categories that the literature indicates likely reflect the underlying mechanisms of strial pathology (as described in the methods): cell morphology [[10]; Table 1], Injury [[1]; Table 2], hearing/hearing loss (Table 3), and upstream regulators [[11]; Table 4]. It is notable that while the transcripts shown represent the significant differences identified from the 23000 mouse genes analyzed represent most of the genes modulated, they are not completely exhaustive. The raw data is provided in the NCBI repository to allow independent analysis by other investigators (see methods for link). For cell morphology (Table 1) most of the genes modulated regulate cell survival, cell signaling, transcriptional regulation, cell adhesion, and ion channels. For injury (Table 2), most of the genes modulated regulate pro-inflammatory cytokines, inflammation, potassium channels, cell signaling, and hypoxia/ischemia. For hearing/hearing loss (Table 3) modulated genes include transporters, intermediary metabolism, and growth factors. For upstream regulators (Table 4), which are all transcription factors, the genes modulated regulate cell signaling, adhesion/migration/differentiation, inflammation/injury response, and of course other transcription factors. There is some overlap within the categories presented in the tables as some of the genes function in multiple pathways as analyzed using the pathway-finder function of the Ingenuity software. What is notable based on the function of the genes listed in Table 2 is that the Alport stria is in an inflammatory state with considerable evidence of injury. A large percentage (approximately 80%) of these genes have never been characterized in the stria vascularis, and thus represent a novel “footprint” for strial pathology. We validated several of these genes using standard qRT-PCR. As shown in Fig 3, most of these genes validated RNA the findings observed by RNA-seq. One notable exception, TNF-α, was significantly up-regulated by RNA-seq, but did not validate by qRT-PCR. This may be due to the fact that TAQman probes only span a single exon which might miss alternatively spliced transcripts. RNA-seq has greater specificity than PCR-based methods, suggesting that the RNA-seq data is the more reliable data set [12].

**Fig 4:**
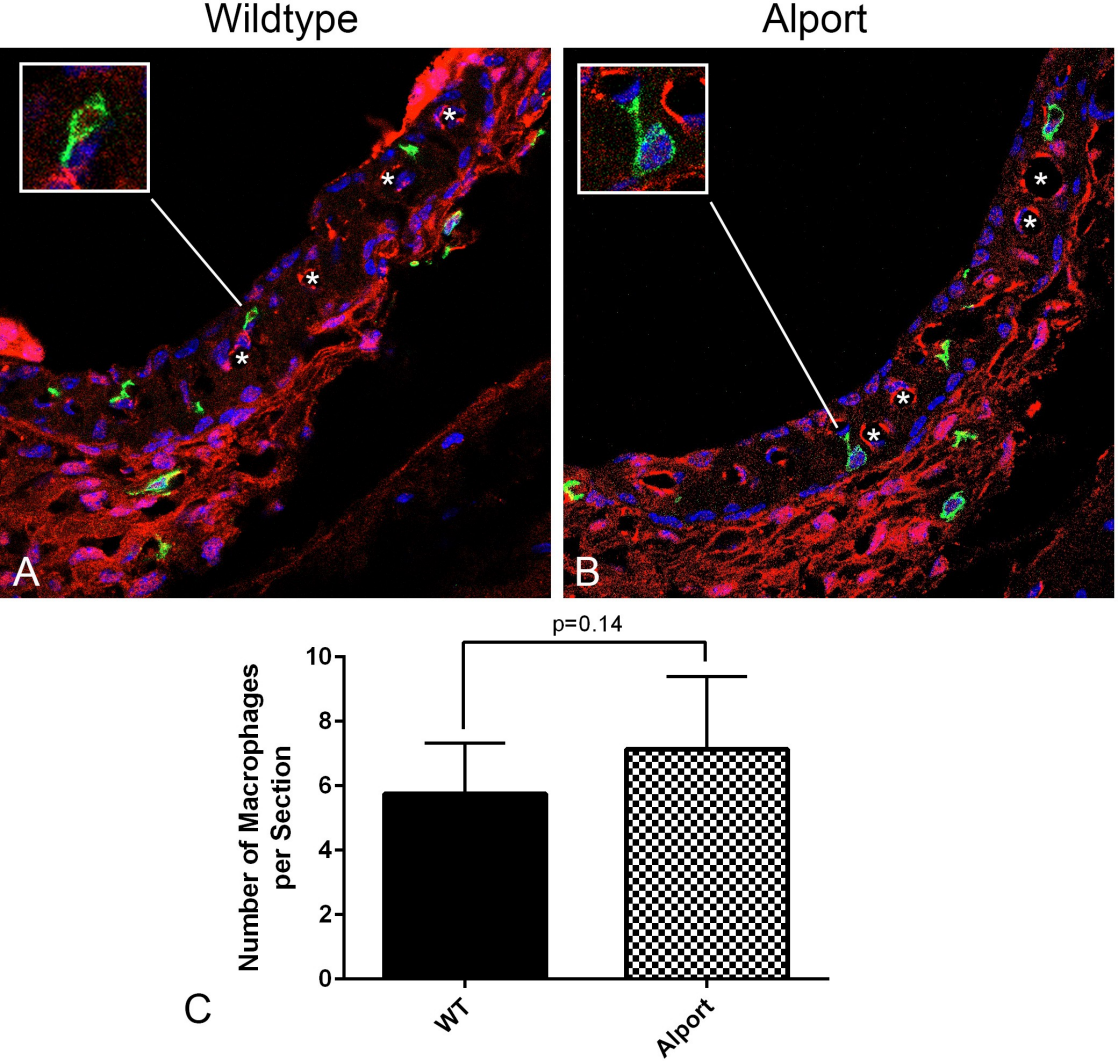
The number of interstitial macrophages in Alport stria versus wild-type stria (7 weeks of age) did not vary significantly, however the macrophages in Alport stria appear activated, with slightly higher numbers, larger cell bodies and numerous cell processes. Mid-modiolar cryosections of cochleae from wild-type (A) and Alport (B) mice were immunostained using anti-desmin (pericyte marker, red) and anti-F4/80 (macrophage marker, green) antibodies. Eight wild-type and 8 Alport mice were analyzed. The data analyzed by two-tailed student’s t-test, but did not achieve significance (C).

It has been previously shown that resident macrophages are activated and non-resident macrophages recruited to the lateral wall and the stria vascularis in response to noise damage, ischemia, or mitochondrial damage [13, 14]. To determine whether the inflammatory cytokines are due to activation/increased numbers of macrophages in the stria vascularis of Alport mice we performed dual immunofluorescence labeling with anti-desmin antibodies (a marker for pericytes) and anti-F4/80 9a marker for macrophages [15], antibodies. The results in Fig 4A show that the number of macrophages in the Alport and wild-type mice appear similar. To validate this observation, macrophages were quantified in mid-modiolar cross sections of the stria from eight wild-type and eight Alport mice (Fig 4B). While the numbers trended higher in the Alport stria, they did not achieve significance.

**Fig 5:**
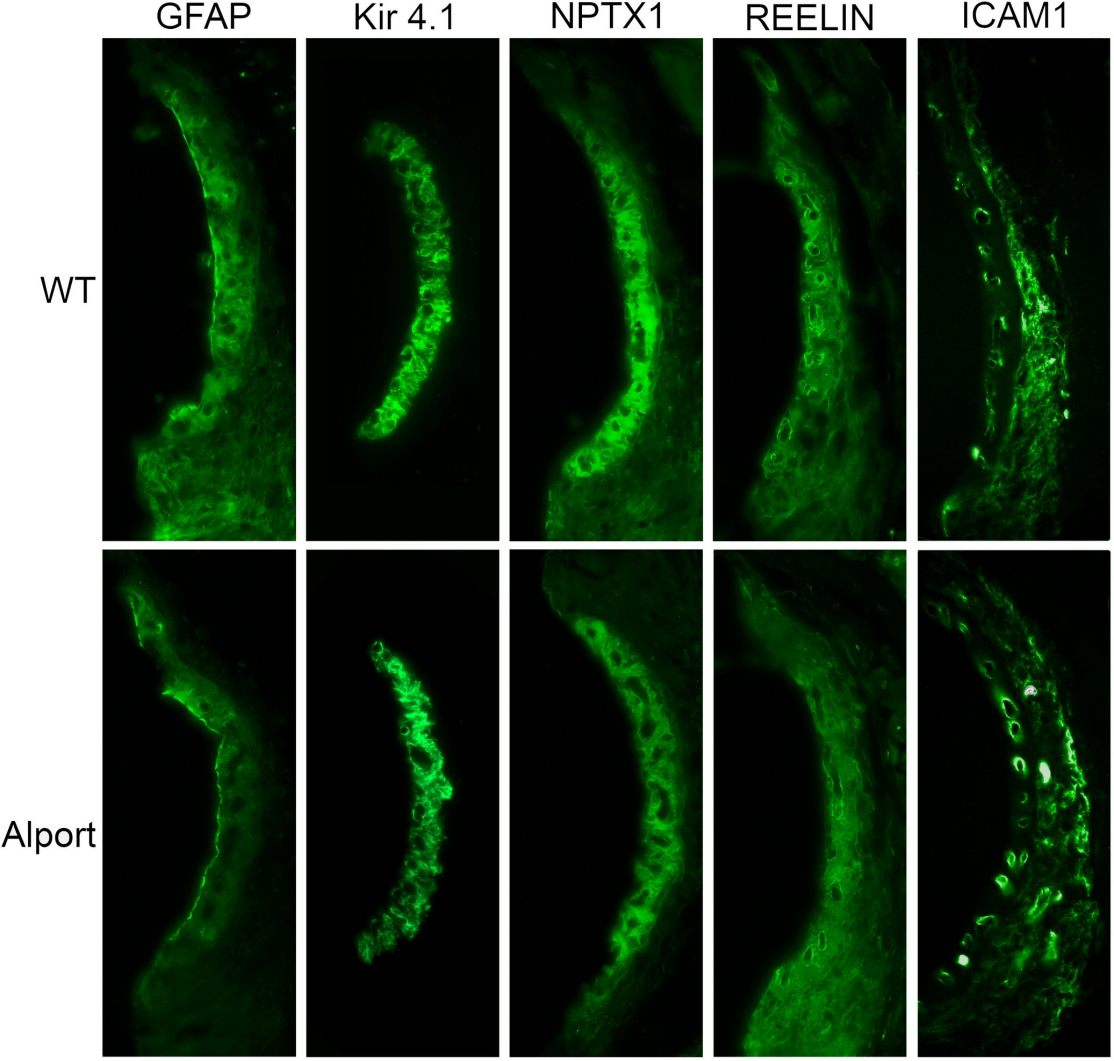
Immunofluorescence analysis for strial expression of proteins encoded for by Gfap, Nptx1, Reelin, and Icam1 genes. To corroborate RNA-seq data, we analyzed protein expression using mid-modiolar cross sections of 7-week-old wild-type and Alport mice. Results show down regulation in Alport compared to wild-type mice for glial fibrillary acidic protein, neuronal pentraxin 1, and reelin, and with up regulation in Alport compared to wild-type for Icam1. All are consistent with the alterations in mRNA expression. We include Kir4.1 as a control for intermediate cell staining [20]. The experiment shown is reflective of four independent experiments using independent groups of animals.

To determine whether the RNA-seq data corroborated with protein expression in the stria, we performed immunohistochemical analysis of four proteins encoded by the genes marked in bold in Tables 2 and 3. Several genes were chosen on the basis that they have never been shown to be expressed in the stria vascularis and thus may reflect novel pathogenic mechanisms. The results in Fig 5 show that a good correlation exists between protein expression and mRNA expression for the four genes/proteins. Glial fibrillary acidic protein [concentrated at the luminal surface of the marginal cells, involved in cell-cell communication; [16]], neuronal pentraxin 1 [localizing to intermediate cells, involved in acute immune response; [17]], and reelin [partially encircling strial vessels, pericyte-like localization, involved in response to tissue injury; [18]] have never been shown to be expressed in the stria, and thus represent novel genes associated with strial pathology. ICAM1, a cell adhesion molecule, has been previously shown to be expressed in strial and spiral ligament vessels [19].

## Discussion

Prior work showed that the SCBMs in Alport mice are thickened relative to age/strain-matched wild-type mice, and that the thickening is associated with an accumulation of extracellular matrix (ECM) [6–8]. As in the renal glomerulus, the mechanism underlying the SCBM thickening is mediated through activation of endothelin A receptors [8, 21]. Blocking these receptors with small molecules prevents accumulation of ECM in the SCBMs and normalizes SCBM thickness ultrastructurally [8]. A cursory look at the resting stria from Alport mice demonstrated that the tissue was in a state of oxidative/metabolic stress, much like the stria from age/strain-matched wild-type mice following noise exposure [8, 22]. Here we extend these findings to demonstrate the full spectrum of changes in gene expression in the Alport stria compared to age/strain-matched wild-type stria. To the best of our knowledge, this is the first application of RNA-seq comparing profiles in normal and diseased stria vascularis.

The results suggest that the stria vascularis is in an inflammatory state with a large number of proinflammatory cytokines and chemokines up-regulated (for example Ccr5, Ccl9, Ccl7, Il17f, and TNF-α) and a number of molecules meant to protect from inflammatory damage are down-regulated (including Ctnf, Ccl3, Il12a, and Smtnl1). A large number of genes are involved in the regulation of the pro-inflammatory NFkappaB response (including Bach2(induced), Card11 (induced), Tnf (induced), Tnfrsf4 (suppressed), and Tmem178 (suppressed)), clearly identifying activation of inflammatory responses in the strial compartment.

That strial function is impacted in Alport mice is evidenced by modulation of a number of transporters and channels that show significant changes in gene expression compared to wild-type littermates. These include Slc2a5, Slc4a4, Slc6a19, Slc8a1, Slc17a8, Slc25a21, Slc26a4, and Spns3. Potassium channels and transport mediators were also affected. For the most part these were significantly down-regulated including Kcna1, Kcnc1, Kcnip 2, Mlc1, and Kcna5. There were exceptions, however, where up-regulation was observed including Kcnc2 and Kcnip1. These channels have not been previously characterized in the stria vascularis, so the consequence of their up regulation is not clear.

Notably, a number of transcription factors are modulated that regulate genes associated with injury and inflammation (Table 4), among them STAT1, NFkappaBIA, FOXa2 and JUNB. These four transcription factors are associated with inflammatory responses and likely contribute to the inflammatory state of the Alport stria vascularis. GATA2 is a transcription factor that regulates transcriptional modulators GATA1 and Klf1, both of which are highly up-regulated in the Alport stria vascularis relative to wild-type, amplifying the transcriptional dysregulation in Alport stria.

As shown in Table 3, several genes are modulated that have been previously shown to be related to hearing loss. Brain-derived neurotrophic factor is markedly down-regulated in the Alport stria relative to wild-type stria. This growth factor has been shown to inhibit spiral ganglion degeneration and thus reduced secretion might compromise cochlear health. Slc26a4 encodes Pendrin, a well-characterized transporter required for regulation of fluid volume in the Scala media [23]. The absence of Pendrin results in deafness. Up-regulation of Pendrin in Alport stria might reflect a compensatory mechanism due to down-regulation of other transporters. FGF10 is required for expansion of the non-sensory regions of the cochlear duct during cochlear development [24]. Whether there is a functional consequence for FGF10 up-regulation (5-fold) in the Alport stria is unclear.

In a recent publication, we showed that the Alport stria was under metabolic stress resulting in elevated expression of hypoxia-related factors [8]. In the current study we provide a more comprehensive profile of strial injury and demonstrate unequivocally that the stria vascularis in the Alport mouse model is in an inflammatory state. As noted above, many of the inflammatory pathways induced in the Alport stria converge at NF-kappaB activation. NF-kappaB has long been known to play a primary role in inflammatory diseases [25]. Therefore, it is of interest to point out that NF-kappaB is induced in the lateral wall of mice subjected to acoustic overstimulation [26]. Exposure of mice to loud noise produces oxidative stress and up-regulation of genes associated with inflammation [1, 22, 27]. The Senescence Accelerated Mouse-Prone 8 (SAMP8 mouse), which shows accelerated aging, shows signs of both inflammation and oxidative stress in the stria vascularis [28]. This likely precedes degenerative changes documented for the strial capillaries in the aging mouse [29]. Collectively, these studies suggest that inflammation associated with strial pathology may be quite common and thus may reflect a more general target to protect against major causes of hearing loss such as presbycusis and noise-induced damage.

Studies of human temporal bones documented splitting of the basilar membrane in the region of the pars pectinata and cellular infilling in the tunnel of Nuel. These investigators concluded that the SNHL associated with Alport syndrome might be associated with abnormal cochlear micromechanics [30, 31]. The Merchant paper further concluded that the SCBMs were not thickened. Careful examination of the data in Merchant et al. it is clear that the SCBMs on the outside of the pericytes are indeed thickened relative to TEM images of normal SCBMs, which is what is observed in the mouse. SCBMs are bilayered, with an internal basement membrane between the endothelial cell and the pericyte and a second basement membrane lining the outer layer of the pericyte [graphically shown in [8]]. The Merchant paper was only considering the endothelial basement membranes, which were indeed of normal thickness. Early studies of human Alport organ of Corti isolated and fixed immediately following death also noted thickening of the SCBMs [32]. Importantly, Moon et al [33] noted significant hearing loss in Alport patients with normal otoaccoustic emissions, an observation that is wholly incompatible with the theory of abnormal cochlear micromechanics, which essentially rules it out. It is quite possible that the splitting of the basement membrane in the pars pectinata is either an artifact of tissue preparation, which can take up to a year for human temporal bones, or occurs long after hearing loss is established.

In summary, the RNA-seq studies presented here show that progressive thickening of the SCBMs in the Alport mouse model is associated with strial inflammation, oxidative stress, and dysregulation of ion channels and transporters. These changes likely account for the sensitivity of Alport mice to noise-induced hearing loss documented earlier [8]. It is important to remember that the Alport SCBMs have a change in the type IV collagen composition [6], which precipitates the progressive changes that culminate in reduced vascular permeability and strial inflammation. It will be of interest to determine the similarities/differences in the inflammatory response for models of presbycusis and noise-induced strial damage. If similar, they may be responsive to more generalized anti-inflammatory therapeutics, as has previously been proposed [13]. Since >80% of these genes have never been described in the stria vascularis, this work provides an important framework for validating therapies aiming to prevent Alport strial dysfunction as well as to define novel molecular pathways associated with strial dysfunction not only in Alport syndrome, but likely other disorders where SCBM thickening has been a noted feature.

## Supporting information

S1 File Copy of vascular permeability-PLOS. (XLSX)

S2 File Copy of gene expression data Dufek et al. (XLSX)

S1 Fig Strial macrophages. (JPG)
