# Supplementary figures and images for: RNA-seq analysis of gene expression profiles in isolated stria vascularis from wild-type and Alport mice reveals key pathways underling Alport strial pathogenesis
Source: PLoS One. 2020 Aug 21;15(8):e0237907. doi: 10.1371/journal.pone.0237907 (PMC7446819; doi:10.1371/journal.pone.0237907)

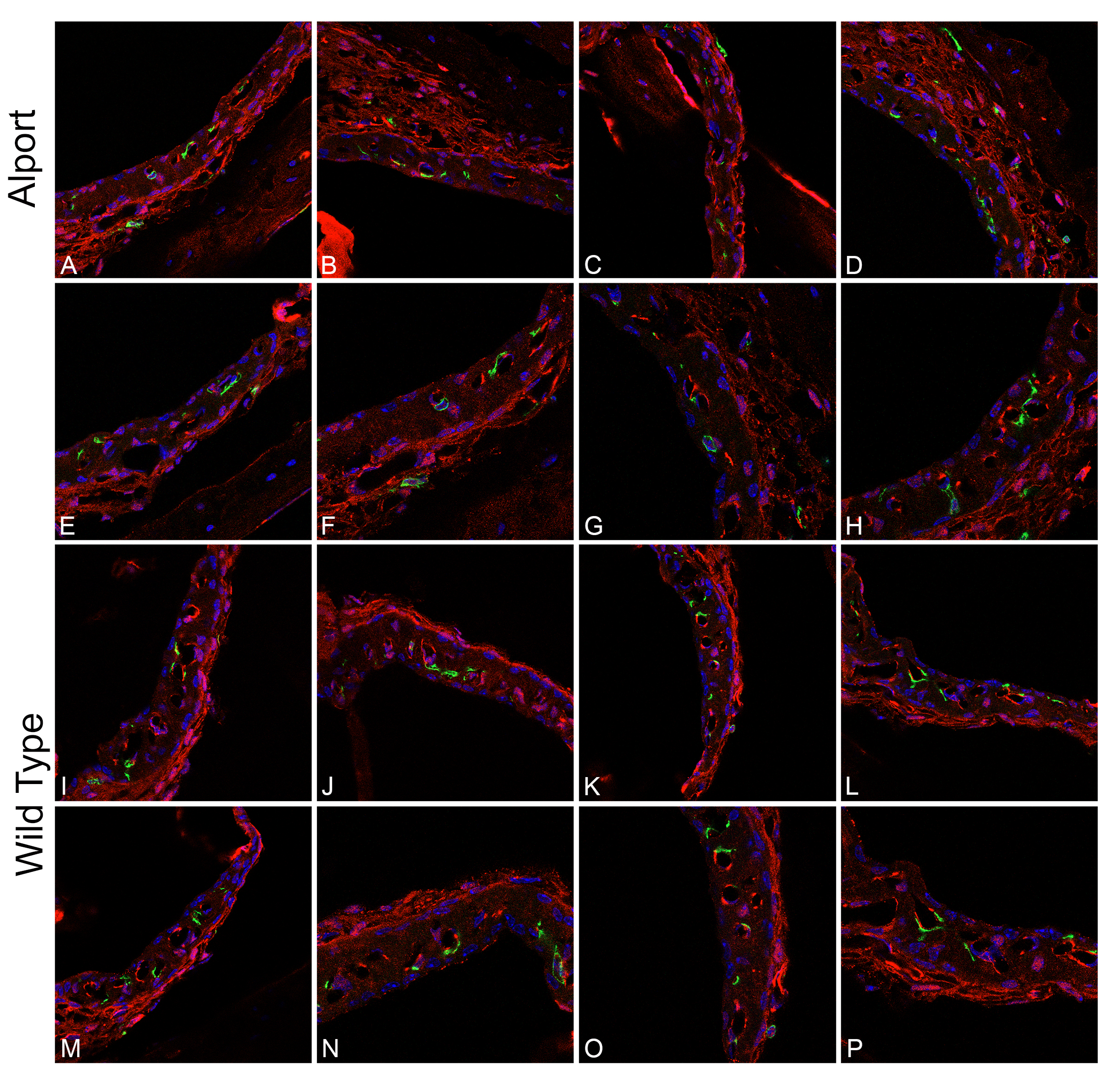

Supplement: S1 Fig — (JPG) [file pone.0237907.s003.jpg]
